# Board social capital and structure, ownership and financial variables of Brazilian companies: A three levels dataset integrating directors, board networks and firm characteristics

**DOI:** 10.1016/j.dib.2019.104502

**Published:** 2019-09-12

**Authors:** Luciano Rossoni, Alex Ferreira Gonçalves

**Affiliations:** Universidade Do Grande Rio, Brazil

**Keywords:** Board interlocking, Director interlocks, Social capital, Social networks, Corporate governance, Ownership structure, Corporate finance, Stock market

## Abstract

This data article incorporates, in an unbalanced panel data, five variables types: financial and market; board structure; board network and social capital; ownership and governance level; the cost of capital. The dataset is formed of 6024 firm-level annual observations based on 622 Brazilian public companies investigated between the years of 2002 and 2015, totaling 56 variables. A three-level data structure was created to allow aggregate directors and network board data into the panel data. Directors' data and adjacency matrix are included to allow for multilevel hierarchical analyzes as well as the use of analytical methods of social networks.

**Table:** Specifications Table

Subject area	*Business, Management, and Accounting*
More specific subject area	*Business, Management, and Accounting (General)*
Type of data	*Panel Data Table*
How data was acquired	*We collected raw data from five sources: B3 Brazilian stock exchange,* Brazilian Securities Commission (CVM), Economatica, Thomson Reuters Eikon, and JP Morgan.
Data format	*Raw, filtered and analyzed.*
Experimental factors	*A sample of companies listed on the Brazilian stock exchange.*
Experimental features	*The panel data incorporates at the firm level five variables types: financial and market; board structure; board network and social capital; ownership and governance level; the cost of capital. Data at the director and network levels are embedded in the data article.*
Data source location	*Brazil*
Data accessibility	*Data are included in this article.*
Related research article	*Goncalves, A. F., Rossoni, L., Mendes-Da-Silva, W.*[1]*. Board social capital reduces implied cost of capital for private companies but not of state-owned companies. Management Decision.*https://doi.org/10.1108/MD-11-2017-1205

**Table:** 

**Value of the Data** • The dataset panel incorporates firm-level variables on board interlock, financial and market indicators, corporate governance, and ownership structure. • The data can be useful for assessing the combined effect of corporate governance, board social capital and networks on financial and market performance on emerging financial markets. • As we organized the data into a three-tiered structure, company, directors, and dyads, it is possible to analyze how the emergence of the board interlock is imbricated with indicators of corporate governance and performance. • As the dataset panel presents a horizon of 14 years with a large number of publicly traded companies, hypotheses about temporal dynamics and structural breaks are possible. • The data are useful for studies of different theoretical perspectives, such as agency theory, network theory, institutional theory, and corporate finance.

## 1 Data

**Fig. 1:**
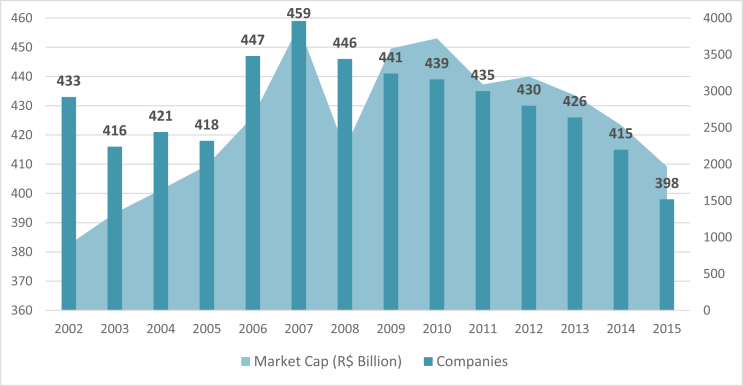
**Brazilian companies investigated annually**. Note: The sum of the market capitalization is represented on the right vertical axis (R$ 1,00 ≅ US$ 0,25).

The unbalanced panel is formed of 6024 firm-level annual observations based on 622 Brazilian public companies investigated between the years of 2002 and 2015. Fig. 1 shows the distribution of the number of companies and the market capitalization annually. We identified all the companies listed on B3 (former BM&F Bovespa), the Brazilian stock exchange, which effectively operated during the analyzed period, regardless of market liquidity.

Data on the Brazilian capital market are very fragmented and not easily collectable. For this reason, the dataset was constructed using five different sources: 1) B3, the only Brazilian stock exchange; 2) Brazilian Securities Commission (CVM); 3) Economatica® databank, an application that brings together the largest amount of market and financial information of Brazilian companies; 4) Thomson Reuters Eikon databank; 5) JP Morgan website www.adr.com. Even using multiple sources, the most relevant variables in the dataset had to be manually collected, filtered and analyzed before they were useful for firm-level analysis. These and all other variables are detailed in the next section.

In the panel data, we have identified 56 variables and attributes of the companies that are available in the supplemental material labeled “1_Panel_Data_Brazilian_Companies”. Data is available in STATA, SPSS and Excel files. To facilitate the understanding of how the variables were operationalized, we divided the description into six blocks: company identification (Table 1); financial and market variables (Table 2); board structure variables (Table 3); board social capital and network variables (Table 4); ownership and governance level variables (Table 5); cost of capital variables (Table 6). In the dataset, the variables are listed in the same order as the tables, with the same name. It should be stressed that not all variables present the same number of cases since many of them depend on market liquidity and analysts' evaluation to be produced. Therefore, in Table 2, Table 3, Table 4, Table 5, Table 6 we list the number of valid cases.

**Table 1:** Company identification.

Variable name	Description
Economatica_Name	Economatica's Company Description (Trading Name)
Company_Name	Corporate Name
Stock_Class	ON: Ordinary shares: grant the voting power at the company's meetings. They are always nominative. ON A: This is a specific class of ordinary shares. To know the characteristics of the class it is necessary to consult the statute of the company. PN: Preferred shares: offer preference in the distribution of results or reimbursement of capital in case of liquidation of the company. PN A, B, C, D or F: These are specific classes of preferred shares. To know the characteristics of each class it is necessary to consult the statute of the company. UNT: A Unit, or certificate of deposit of shares, is not a stock, but rather a “package” of asset classes, which can be formed by common shares, preferred shares, and subscription bonuses. UNT N2: This is a Unit that belongs to governance level 2.
Company_status	Company status in the Stock market in 2017, December.
Stock_Exchange_Company_Code	The code used in the Stock Market trading process. Composed of four letters related to the company name and a number that identifies the type of the share as follows: 1 - the right to subscribe for a common share; 2 - the right to subscribe for a preferred share; 3 - common share; 4 - preferred share; 5, 6, 7, 8 … - preferred shares that belong to different classes; 9 - subscription receipt for common shares; 10 - subscription receipt for preferred shares; 11 - Units and BDRs (Brazilian Deposit Receipts - Certificates of deposit of shares of foreign companies); 33 - BDRs (Brazilian Deposit Receipts) Governance Level 3.
CNPJ	Brazilian national registry number of legal entity.
Economatica_Code	Company identification number used by Economatica Software to identify each company.
CVM_Code	Brazilian Securities Commission (CVM) unique identification code
Industry	Area of companies' activity listed on the stock market.
Year	Indicates the year in which the variables are measured, acting as the basis of time measurement.

Source: Economatica®.

**Table 2:** Financial and market variables.

Variable name	Data type	Description	Cases
Total_Assets	Real-valued numeric	Book value of Total Assets adjusted for inflation. Amounts in thousands of reais (R$).	6024
Volatility	Real-valued numeric	The calculation of the annual volatility related to the stock earnings is based on the last 12 months, adjusted for inflation. It indicates the intensity and the frequency of the oscillations in the price of an asset in a certain period, considering the series of stock daily quotations. It is the degree of variation of the share price in a given period of time, the greater the volatility the greater the risk.	2068
Liquidity	Percentage	Liquidity is the facility that stocks have to be converted into cash and can be measured by the daily volume of trades made with them. For a stock to have high liquidity, it is necessary that there be a frequent presence in the trading sessions, and simultaneously in the period with a high volume of negotiations and a high number of trades.	6024
		Stock Liquidity = 100 * w/P * sqrt (n/N * v/V) at where:	
		p = number of days in which there was at least one business with the share within the chosen period	
		P = total number of days in the chosen period	
		n = number of deals with the share within the chosen period	
		N = number of businesses with all shares within the chosen period	
		v = cash volume with the share within the chosen period	
		V = cash volume with all shares within the chosen period	
ROA	Real-valued ratio	Return on Asset (ROA): The ratio between the company's profitability and the total volume of its assets. It is the company's ability to use its assets to generate profit. The data was consolidated annually (2002–2015), with reference month set in December of each year. The ROA is also known as LAJIRDA, which means earnings before interest, income tax, depreciation and amortization on total assets. This measure is obtained through the equation:	5976
		Ln (ROA) = Ln (LAJIR/AT), wherein:	
		• LAJIR = profit before interest and taxes;	
		• AT = book value of total assets.	
Market_Value	Real-valued numeric	Market value is the amount that stock market investors are willing to pay for trading on stock exchanges related to a specific company. It is obtained by multiplying the unit value of shares by the total number of shares that make up capital stock. The amounts are presented in thousands of reais (R$), adjusted by the inflation indexes.	4228
Beta	Real-valued numeric	The Beta Index is an indicator that measures the sensitivity of an asset to the behavior of a portfolio that represents the market. It is a measure of the risk that an investor is exposed to when investing in a particular asset compared to the whole stock exchange market.	3375
		Beta = (Covariance between Return on Asset and Market)/(Variance of Return on Market)	
		Beta High: Beta> 1	
		Beta Neutral: Beta = 1	
		Beta Low: Beta <1	
Current_Liabilities	Real-valued numeric	Current liabilities are obligations normally paid within one year: accounts payable, debts with suppliers of goods or raw materials, taxes to be collected (for the government), bank loans due in the next 360 days, provisions (expenses incurred, generated, not yet paid but already recognized by the company: income tax, vacation, 13th salary, etc.). Values consolidated annually and adjusted for inflation. Data in thousands of reais (R$).	6017
Long_Term_Liabilities	Real-valued numeric	Long-term liabilities are debts of a company which will be settled after the end of the following financial year. In most institutions, the “year” is considered a calendar year. Examples are financings, bills to pay, among others. Values consolidated annually and adjusted for inflation. Data in thousands of reais (R$).	3933
Floating_Asset	Real-valued numeric	Floating assets are a reference to assets and rights that can be converted into cash in the short term. Assets that may be considered as current assets include cash, bank account, financial investments, accounts receivable, inventories, prepaid expenses, bank deposits, commodities, commodities, and securities. Values consolidated annually and adjusted for inflation. Data in thousands of reais (R$).	6017
Stock	Real-valued numeric	Stock refers to all tangible assets held for sale or own use in the ordinary course of business, goods in the process of production for sale or for own use or that are intended for consumption in the production of goods for sale or own use. Values consolidated annually and adjusted for inflation. Data in thousands of reais (R$).	5613
Accounting_Asset	Real-valued numeric	Accounting Asset is the set of tangible assets and rights used to achieve the entity's activities-purposes. The assets are necessary for the development of the company's corporate purpose, such as real estate, furniture, and utensils, installations, machinery and equipment, land vehicles, air, sea and rail, among others. Values consolidated annually and adjusted for inflation. Data in thousands of reais (R$).	6022
Revenue	Real-valued numeric	Revenue is the proceeds from the sale of goods or the provision of services. Values consolidated annually and adjusted for inflation. Data in thousands of reais (R$).	5571
Tobin_Q	Real-valued ratio	The simplified Chung and Pruitt [3] indicator measure the performance of a particular company based on the sum of the market value of its shares, plus its debts, which is divided by the book value of its total assets.	4228
		Tobin Q = (VMaO + VMaP + (VCPC - VCAC + VCE + VCDLP))/VCAT	
		VMaO = (Market_Value field);	
		VMaP = (Market_Value);	
		VCAT = (Total_Assets);	
		VCPC = (Current_Liabilities);	
		VCAC = (Floating_Assets);	
		VCE = (Stocks);	
		VCDLP = (Long_Term_Liabilities).	
Assets_Tangibility	Percentage	It is the ratio of Fixed Assets (Stock plus Accounting_Asset) divided by Total_Assets.	5610
Sales_Increasing	Percentage	Percentage growth of revenue of one year in relation to the previous year.	4622
		Consolidated annual sales amount (in thousands of reais). In order to define the sales growth of 2002, it was necessary to collect data for 2001, even though it was outside the period determined for this study. The variable was operated using the formula:	
		Sales Growth (t-1) = Sales Volume (t-1) - Sales Volume (t))/(Sales Volume (t-1))	

Source: Economatica®.

**Table 3:** Board structure variables.

Variable name	Data type	Description	Cases
Board_Size	Count	It consists of the number of board members of company i, in a year t.	6005
Outsiders_Board_number	Count	Number of directors who do not play roles other than the role of directors. External directors are those who sit on the board of directors and do not hold management positions within the company but may be linked to groups of shareholders (mainly controlling shareholders). Also included in this group are the so-called independent directors, who are those who have no connection with management positions or shareholders.	6005
Duality	Binary	On boards with a dual structure, the CEO occupies two positions (CEO and Chairman of the board) simultaneously. The data was filtered by the director's function code (code 30 - chairman of the board and president director).	6005
CEO_in_Board	Binary	Participation of the CEO on the board in positions other than of chairman of the board. A filter was performed by the member function code, filtering codes 31 (vice president of the board and president-director), 33 (effective director and president-director) and 36 (substitute director and president-director).	6005
Busy_Directors	Count	Busy directors are those who occupy simultaneously seats on various boards, commonly called in the literature by the expression “busy directors”. We consider that those who occupy 3 or more positions on different boards are classified as “busy directors”.	6005
Busy_Board	Binary	They were classified as busy boards those boards in which more than 50% of its members participate in 3 or more different boards (busy directors).	6005

Note: Aggregated data from the dataset “3_Directors_Data”, available at supplementary material.

**Table 4:** Board social capital and network variables.

Variable name	Data type	Description	Cases
Degree	Count	Degree or Board Interlock refers to the number of companies linked by the directors that one company shares with other companies. Thus, the centrality corresponds to the number of board members on a board who simultaneously hold positions on boards of other companies, regardless of whether the companies belong to the same controlling group or not.	6016
EigenVector	Continuous	Alpha de Bonacich (Eigenvector): Measure that evaluates the degree of centrality of a company not individually, but also considers the centrality of neighbors (councils) to compose the indicator. It conceives a hierarchy between loops with greater or lesser centralization in the network. This measure, in addition to the adjacent ties, considers the centrality of these ties, which makes it different from the Centrality of Degree. Measurement operationalized through the Eigenvector feature of the UCINET® software.	6016
Coefficient_Cluster	Percentage	Indicator that measures the local density of the network and indicates how the contacts of an actor are recursively linked together. In other words, the greater the number of clicks they form (mutual ties between at least 3 participants), the larger the grouping of the network. This indicator ranges from 0 to 1, where the coefficient “0” indicates fully ungrouped networks while the coefficient “1” indicates fully clustered networks. This measure will be calculated for each year of the company's participation in the stock exchange.	6016
Efficiency	Percentage	Burt's measure of the effective size of the ego's network (essentially, the number of alters minus the average degree of alters within the ego network, not counting ties to ego). Considering that an actor i can present n contacts, we must: Efficiency = [EffSize/n] = [(Dalters - n)/n] = {[(2 l/n) - n]/n} On what: • Efficiency = Proportion of non-redundant loops; • Dalters = Redundant contacts; • n = alters; • l = Number of ties between n (alters).	6016
Social_Capital (Thousands_of_Reais)	Continuous	Resources immersed in the network of social relations between Boards of Directors through the so-called board's interlocks, accessed or/and mobilized in intentional actions through the interaction of the network and the resources present in it [4]. Social capital is constituted by the set of relations that give an individual access to resources that do not necessarily belong to him and that he would not have access were it not for their relationships. Access is carried out through so-called boards interlocks, in which a network rich in social capital is a network rich in mobilizable resources. The operationalization of Social Capital is given by the sum of the relational resources present in direct relationships. The ties of the ego network were identified, that is, the direct ties of each company with the others through the board interlocks. Soon after, the market value of each of the company's relationships (total value of the shares traded on the stock exchange) was identified. Finally, the value of the relational resources of each tie of the company was added. The financial data was collected on Economatica software. Values in thousands of Reais (R$).	6016
Heter_Social_Capital (Thousands_of_Reais)	Continuous	To reach indirect relations, we performed the structural holes procedure, which generated the redundancy value of each direct relation in relation to each of the network companies. The concept of redundancy is based on the following principle: If A is connected to B and C, and B is connected to C, the tie from A to B is redundant, because actor A can influence B through C. The measure of redundancy calculates, for each actor, how much of the other actors in the network are also connected to another actor. To say that the tie from A to B is highly redundant means that most of the other actors in the network also have a tie with B. Actors in networks with high redundancies are actors that are in networks with few “structural holes.” We then reduce the value 1 of the redundancy value found for each company (of each Alter), thus generating a heterogeneity score for each alters. The greater the heterogeneity, the greater the number of structural holes present in the network. As the last step, this heterogeneity value was multiplied by the market value of each existing tie. Finally, the value of the relational resources of each tie of the company was added. The financial data was collected on Economatica software. Values in thousands of Reais (R$).	6016

Source: Brazilian Securities and Exchange Commission (CVM), Reference Forms and Annual Information (IAN).

Note: Network variables generated from adjacency matrices “2.1_Board_Social_Capital_Network_Data” and “2.2_Board_Heterogeneous_Social_Capital_Network_Data”, available at supplementary material. Variables processed by the UCINET® software.

**Table 5:** Ownership and governance level variables.

Variable name	Data type	Description	Cases
ADR_Name^12^	Nominal	American Depository Receipt (ADR) are certificates that represent the shares of a foreign (non-US) company traded on the New York or NASDAQ stock exchanges. In addition to making it easier for Americans to invest in foreign companies without worrying about exchange rate changes, foreign exchange trading rules, and language, ADRs serve as indicators of a higher level of governance that are associated with lower capital costs. This variable comes from the cross-data of the CVM database with JP Morgan through the website www.adr.com.	419
ADR_Level^12^	Nominal	Identifies the Level of ADR. It should be noted that the ADRs classified as Levels 2 and 3 were used. Levels 1 and N144A were not considered, since there is no requirement to register with the Securities and Exchange Commission (SEC) or to comply with the Generally Accepted Accounting Principles (GAAP), thus having greater risk, making it difficult to compare them with other investments due to the difference in accounting. It is also important to highlight the basic difference between Level 2 and Level 3 ADRs. Level 2 requires partial compliance with GAAP and Level 3 requires full attendance. We emphasize that only Level 2 and 3 ADRs can be listed on the New York Stock Exchange, the American Stock Exchange or NASDAQ. This variable comes from the cross-data of the CVM database with JP Morgan through the website www.adr.com.	419
Bovespa_Classification³	Nominal	Refers to B3 governance levels, namely: “New Market”, Level 2, Level 1, “Bovespa Plus” and “Traditional” market.	6024
Percentual_Biggest_Owner_ON^4^	Percentage	The largest shareholder's ownership of the company's voting shares.	4275
Sum_3_Largers_Owners_ON^4^	Percentage	Sum of the three largest shareholder's ownership of the company's voting shares.	4275
Sum_5_Largers_Owners_ON^4^	Percentage	Sum of the five largest shareholder's ownership of the company's voting shares.	4275
Percentual_Biggest_Owner_PF	Percentage	The largest shareholder's ownership of the company's preferred shares.	2614
Sum_3_Largers_PF^4^	Percentage	Sum of the three largest shareholder's ownership of the company's preferred shares.	2614
Sum_5_Largers_PF^4^	Percentage	Sum of the five largest shareholder's ownership of the company's preferred shares.	2614
HHI_3_Largers_ON^4^	Ratio	The concentration of property is measured by means of an adaptation of the Herfindahl-Hirschman Index (HHI). This index is usually used to measure the degree of competition in a particular industry, but it is also used as a measure of the concentration of ownership in a given company. Their values range from 0 to 1, where the higher the index, the higher the concentration. It is calculated by summing the square of the individual voting shares owned by the three largest shareholders. It is defined as HHI: HHi = ∑_(i=1)_(β_i_^2^) On what: β_i_ = Q_i_/(Σ_(i=1)_ Q_i_), is the ownership percentage of owner i.	4275
HHI_5_Largers_ON^4^	Ratio	The concentration of property is measured by means of an adaptation of the Herfindahl-Hirschman Index (HHI). This index is usually used to measure the degree of competition in a particular industry, but it is also used as a measure of the concentration of ownership in a given company. Their values range from 0 to 1, where the higher the index, the higher the concentration. It is calculated by summing the square of the largest individual voting shares owned by the five largest shareholders. It is defined as HHI: HHi = ∑_(i=1)_(β_i_^2^) On what: β_i_ = Q_i_/(Σ_(i=1)_ Q_i_), is the ownership percentage of owner i.	4275
HHI_3_Largers_PF^4^	Ratio	The concentration of property is measured by means of an adaptation of the Herfindahl-Hirschman Index (HHI). This index is usually used to measure the degree of competition in a particular industry, but it is also used as a measure of the concentration of ownership in a given company. Their values range from 0 to 1, where the higher the index, the higher the concentration. It is calculated by summing the square of the individual preferred shares owned by the three largest shareholders. It is defined as HHI: HHi = ∑_(i=1)_(β_i_^2^) On what: β_i_ = Q_i_/(Σ_(i=1)_ Q_i_), is the ownership percentage of owner i.	2614
HHI_5_Largers_PF^4^	Ratio	The concentration of property is measured by means of an adaptation of the Herfindahl-Hirschman Index (HHI). This index is usually used to measure the degree of competition in a particular industry, but it is also used as a measure of the concentration of ownership in a given company. Their values range from 0 to 1, where the higher the index, the higher the concentration. It is calculated by summing the square of the individual preferred shares owned by the five largest shareholders. It is defined as HHI: HHi = ∑_(i=1)_(β_i_^2^) On what: β_i_ = Q_i_/(Σ_(i=1)_ Q_i_), is the ownership percentage of owner i.	2614
Class_Ownership^1^	Nominal	This field identifies whether the company is: National Private, Foreign Private or Public. This identification was made for each year of the survey, that is, from 2002 to 2015, since some companies changed ownership type during the analyzed period. For the definition of the type of property, we use the common shares, that is, those that give right to the vote. The definition for the classification of a company as “foreign” used the criterion of the Brazilian Central Bank and several international organizations, which considers the company as foreign when more than 10% of its shares are controlled by foreign capital. The companies classified as Public comprise exclusive public companies and mixed capital companies where the State has share control.	6024

Source: ^1^ Brazilian Securities and Exchange Commission (CVM); ^2^ JP Morgan; ³ B3 Stock Exchange; ^4^ Economatica®.

Note: Concentration of property variables produced by the authors.

**Table 6:** Cost of capital variables.

Variable name	Data type	Description	Cases
Cost_of_Capital (MEAN)_RPEG_Ex_Ante	Ratio	Cost of Capital R_PEG_: Proxy obtained according to Price-Earnings to Growth model. Easton [5] defines RPEG capital cost as: Pt = (EPS _(t + 2)_ - EPS _(t + 1)_)/(R_PEG_)^2^ On what: R_PEG_: Ex-ante Capital Cost at date t, where PEG refers to the price-earnings-growth model of Ohlson and Juettner-Nauroth [6] (Price-Earnings to Growth ratio); EPS _(t + 2)_: (earnings per share) Average value of accounting profit projected by analysts at t + 2. EPS means the net earnings per share obtained by the company in a given period t; EPS _(t + 1)_: (earnings per share) Average value of accounting profit projected by analysts at t + 1; Po = Current share price on the last day of the quotation for the year for which the cost of equity (in t_0_) is being calculated. For the calculation, according to the equation presented, the cost of capital Ex-Ante in December 2011, for example, is calculated from the estimated EPS for the end of 2012 and 2013.	814
Cost_of_Capital (MEAN)_RPEF_Ex_Ante	Ratio	The Ex-Ante R_PEF_ cost of capital data was collected from the Thomson Reuters Eikon platform and found in the database in decimal values. According to Espinosa and Trombetta [7], the cost of capital R_PEF_ is defined by the equation: Po = [EPS_(t + 1)_ + R_PEF_dps_(t + 1)_ + EPS_(t + 2)_]/[(R_PEF_ + 1)^2^–1] On what: R_PEF_: Ex-Ante Capital Cost at date t, where PEF stands for a prospective price-earnings model (Price to Foward Earnings Model); EPS _(t + 2)_: (earnings per share) Average value of accounting profit projected by analysts at t + 2; EPS _(t + 1)_: (earnings per share) Average value of accounting profit projected by analysts at t + 1; dps _(t + 1)_: Dividend per expected share at t + 1; Po = Current share price on the last day of the quotation for the year for which the cost of equity (in t_0_) is being calculated.	847
Cost_of_Capital (MEAN)MPEG_Ex_Ante	Ratio	Proxy obtained according to the model Modified Price to Earnings Growth model. According to Easton [5], the formula for determining the cost of capital R_MPEG_ is: Po = [EPS_(t + 2)_ + R_MPEG_.dps_(t + 1)_ - EPS_(t + 1)_]/(R_MPEF_)^2^ On what: RMPEG: Ex-ante Capital Cost at date t, where MPEG stands for Modified Price to Earnings Growth model; EPS _(t + 2)_: (earnings per share) Average value of accounting profit projected by analysts at t + 2; EPS _(t + 1)_: (earnings per share) Average value of accounting profit projected by analysts at t + 1; dps _(t + 1)_: Dividend per expected share at t + 1; Po = Current share price on the last day of the quotation for the year for which the cost of equity (in t_0_) is being calculated.	503
Cost_of_Capital (MEAN)_WACC_Ex_Post	Ratio	Cost of Capital WACC (Weighted Average Cost of Capital - Ex-Post): Weighted average cost of equity and third-party capital. In terms of the coefficient, the cost of capital represents a minimum rate that the company must obtain in its operations, which indicates the minimum necessary remuneration to be earned to maintain the value of its shares and the respective sustainable growth of the company. This way of measuring the cost of capital is called Ex-Post and assumes the following expression [8]: WACC = (E/V × Re) + [D/V × Rd × (1-Tc)] On what: WACC = Weighted Average Cost of Capital (WACC); Tc = rate of income tax and social contribution of the legal entity; E = market value of equity (company) or shareholders' equity (in R$); D = market value of the company's third-party capital (in R$); V = E + D (market value of total capital, in R$); E/V = ratio of equity to the total financing of the company (in market values); D/V = proportion of third-party capital over the total financing of the company; Re = Equity Rate - Capital Assets Pricing Model or Capital Asset Pricing Model (CAPM). CAPM = Risk free rate + [Beta * country risk premium]; Rd = cost rate of third-party capital before income tax. The cost of third-party capital is defined in accordance with the onerous liabilities identified in the loans and financing maintained by the company. It is calculated using consolidated government rates, debt adjustment factor and the ratio of long- and short-term debt to total debt. Rd = ((Short-term debt) * Short-term debt pre-charge + Long-term debt * Long-term debt pre-charge)/Total debt) * (1-Tc).	2628

Source: Thomson Reuters Eikon.

Note: Cost of capital variables produced by authors.

## 2 Experimental design, materials, and methods

**Fig. 2:**
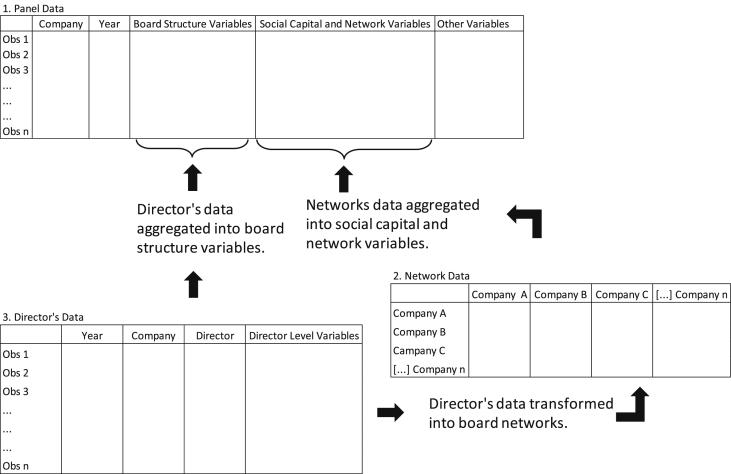
Relationship between three level datasets.

Due to the large number of variables, whose experimental design is different for each one of them, we specify in the description column of Table 1, Table 2, Table 3, Table 4, Table 5, Table 6 how they were operationalized. Board structure variables (Table 3) and social capital board and network variables (Table 4) have a more complex design. For this reason, we use two auxiliary datasets. In Fig. 2, we represent how each of the datasets relate to composing the panel data variables.

To build the board structure variables, we need data about the directors in each of the years. Director-level data is available in the “3_Directors_Data” dataset, which points out the directors at each company, as well as some attributes that were essential in defining the board structure (total of 67,957 records).

We generated the board network and social capital variables also from the directors' level data. First, we conceived an affiliation matrix in a 2-mode format for each year studied, crossing companies in one mode with directors in another. Then, through the UCINET® software, this 2-mode matrix was converted into a 1-mode matrix at the company level. In such an adjacency matrix, two companies are directly linked if they shared at least one director (board interlocking). Therefore, the presence of ties was defined in the binary form in the matrix cells. We then used UCINET® software to generate relational variables at the company level, later aggregated into panel data (see Table 4 for details). The adjacency matrices valued for each year are in the supplementary material: “2.1_Board_Social_Capital_Network_Data”, whose value greater than 0 represents the market value of a company alter (column) in relation to a particular company (ego); the adjacency matrix valued “2.2_Board_Heterogeneous_Social_Capital_Network_Data” considers the same market value of the company alter, but weighted by the heterogeneity of the ties. Details on the board social capital measure can be seen in Rossoni, Aranha and Mendes-da-Silva [2].

## Conflict of interest

The authors declare that they have no known competing financial interests or personal relationships that could have appeared to influence the work reported in this paper.
